# Selective magnetic particle imaging of CD44 expressing cells using iron oxide nanoprobes functionalized with chemically modified hyaluronan

**DOI:** 10.1039/d5na00979k

**Published:** 2026-06-16

**Authors:** Mohammad H. El-Dakdouki, Chia-wei Yang, A. K. M. Atique Ullah, Fei Fan, Kunli Liu, Baraah U. Hijazi, Xuefei Huang

**Affiliations:** a Department of Chemistry, Faculty of Science, Beirut Arab University P.O. Box 11-5020, Riad El Solh 11072809 Beirut Lebanon m.eldakdouki@bau.edu.lb; b Department of Chemistry, Michigan State University 578 S. Shaw Lane East Lansing MI 48824 USA huangxu2@msu.edu; c Institute for Quantitative Health Science and Engineering, Michigan State University East Lansing MI 48824 USA; d Department of Biomedical Engineering, Michigan State University East Lansing MI 48824 USA

## Abstract

Hyaluronan (HA) is a naturally occurring biocompatible and non-immunogenic linear anionic glycosaminoglycan. In an attempt to enhance the selective binding of HA to the cluster of differentiation 44 (CD44), we recently identified a novel chemically modified HA derivative (referred to as G2HA) with improved affinity to CD44 compared to the native HA polymer. In this study, we aimed at conjugating G2HA to iron oxide nanoparticles (IONPs) and assessing the uptake of the G2HA-IONP nanocomposite by CD44 expressing cells, namely 4T1 breast cancer cells. In addition, the utility of G2HA-IONPs as efficient contrast agents for Magnetic Particle Imaging (MPI) was studied. The IONPs were synthesized by the thermal decomposition method, and G2HA was conjugated to the nanoparticles by the ligand exchange method. HA-IONPs were prepared similarly for comparison. The uptake of G2HA-IONPs by 4T1 breast cancer cells was HA-receptor dependent, and higher than that of HA-IONPs at the same iron concentration as validated in multiple assays. The cell viability assay verified that G2HA-IONPs were biocompatible at the highest tested dose. The suitability of G2HA-IONPs as MPI contrast agents was tested. MPI of cells that received the nanoparticles demonstrated that the improved uptake of G2HA-IONPs translated into more intense signals, with a 2.4-fold MPI signal enhancement compared to those from cells that received HA-IONPs. Overall, the findings of this study validated the role of the modified HA derivative (G2HA) in enhancing the uptake of iron oxide nanoparticles and asserted the potential applicability of the developed G2HA-IONPs for selective imaging of CD44-expressing cells and as a potential vehicle for drug delivery.

## Abstract 2

Modified hyaluronan coated iron oxide nanoparticles as efficient MPI contrast agents.

## Graphical abstract

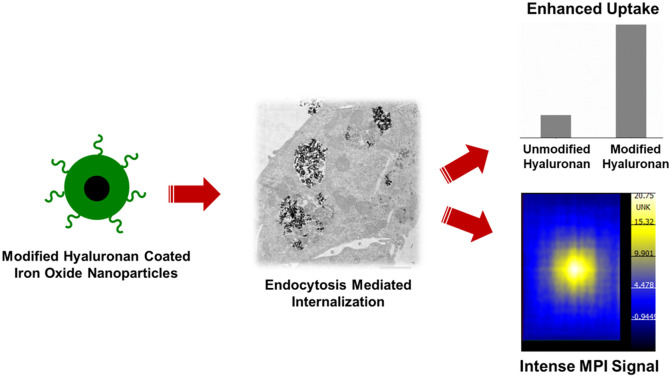

## 1 Introduction

Magnetic Particle Imaging (MPI) is an emerging non-invasive imaging modality that combines the high spatial and temporal resolutions of ultrasound, MRI, and CT, the high sensitivity of PET and SPECT, and the ability to track and quantify magnetic tracers, all while remaining more cost-effective.^1–3^ MPI generates highly sensitive and quantitative three-dimensional (3D) images in real time by directly detecting a magnetic tracer (contrast agent) rather than the surrounding tissues, which appear transparent to MPI because of their diamagnetic properties.^4^ Superparamagnetic iron oxide nanoparticles (SPIONs) are the preferred contrast agents for MPI because of their superparamagnetism, biocompatibility, ease of preparation and surface functionalization, and stability in different media.^5–8^ Despite being an emerging technology, MPI holds great promise in the medical diagnostics sector.^9^ However, clinical maturation necessitates the development of improved imager design, enhanced tracer performance, and standardized imaging protocols. In fact, the practical implementation of MPI is strongly influenced by the intrinsic^10^ and extrinsic^11–14^ properties of the contrast agent. The generation of reproducible high-resolution images by MPI necessitates the fabrication of superparamagnetic magnetic nanoparticles with uniform size and size distribution. In this regard, our recent research has focused on the development of magnetic nanoprobes for enhanced MPI contrast and dual modality imaging in cancer models.^15,16^

To facilitate enrichment of SPIONs at the desired sites, cell surface receptors can be selectively targeted by ligand modified-SPIONs. The cluster of differentiation 44 (CD44) is a receptor that has critical implications in the pathogenesis and progression of diseases such as cancer and atherosclerosis.^17–20^ The natural polysaccharide, hyaluronan (HA), is the principal endogenous ligand for CD44.^21^ HA is a vital constituent of the extracellular matrix and synovial fluid, and it mediates vital biological processes, including cell differentiation, wound healing, proliferation, and migration.^22,23^ While CD44 is expressed in low levels on some normal cells, it exists in a quiescent state with low binding affinity to HA, and requires regulated activation before binding to HA. However, CD44 on cancer cells exists in an activated state with high binding affinity to HA due to alternative splicing and posttranslational modifications.^24,25^ This renders CD44 an interesting and viable target for selective targeted therapy and imaging of tumors while preserving normal tissue. To enhance HA's affinity and selectivity to CD44, we recently reported the preparation of a combinatorial library of HA derivatives using the Ugi reaction and identified a lead analogue (G2HA) with significant enhancement in binding to CD44 using surface plasmon resonance. The enhanced binding translated into improved uptake by cancer cells.^26^

Herein, we report the development of G2HA-coated iron oxide nanoparticles (G2HA-IONPs) as selective MPI contrast agents for targeted imaging and detection of CD44 expressing cancer cells. IONPs were synthesized *via* the thermal decomposition method, and HA or G2HA were anchored onto the surface of the nanoparticles by the ligand exchange method. The targeted nanoformulations were thoroughly characterized by various analytical techniques to assess size, size distribution, surface charge, morphology, and magnetic properties. Various uptake studies demonstrated the role of the modified HA polymer in promoting the accumulation of nanoparticles in 4T1 breast cancer cells, yielding considerably higher contrast in MPI. The findings of this study will have tremendous impacts towards developing new MPI contrast agents for targeted imaging of CD44-mediated diseases such as cancer.

## 2 Materials and methods

### 2.1 Reagents and instrumentation

Reagent grade chemicals were used in this study as supplied by the manufacturers. Sodium hyaluronate (average molecular weight of 31 kDa) was obtained from Lifecore Biomedicals. 1,2-Hexadecanediol, nuclear fast red solution, benzyl ether, iron(iii) acetylacetonate, fetal bovine serum (FBS), and ethyl-(3,3-dimethylaminopropyl) carbodiimide hydrochloride (EDCI) were obtained from Sigma-Aldrich. Mallinckrodt provided potassium ferrocyanide [K_4_Fe(CN)_6_] trihydrate, Acros Organics delivered *n*-hexanol and adipic dihydrazide, and Fluka supplied oleylamine. CellTiter 96®Aqueous One Solution, containing 3-(4,5-dimethylthiazol-2-yl)-5-(3-carboxy-methoxyphenyl)-2-(4-sulfophenyl)-2*H*-tetrazolium, inner salt (MTS) and supplemented with phenazine ethosulfate (PES) as an electron coupling reagent, was acquired from Promega. Dialysis tubing (MWCO of 3500 Da) was procured from BioDesign Inc. Pen/strep mixture, sodium pyruvate (100 mM), glutamine, phosphate-buffered saline (PBS), and fluorescein isothiocyanate (FITC) were sourced from Invitrogen. 4T1-Luc2 mouse epithelial breast cancer cells (ATCC CRL-2539-LUC2) and MCF-7 epithelial human breast cancer cells (ATCC HTB-22) were cultured in RPMI 1640 medium (Gibco™) and DMEM (Gibco™), respectively. Both media were supplemented with 1 mM sodium pyruvate, 1% pen/strep mixture, and 10% FBS. Cells were incubated under standard culture conditions of 5% CO_2_ in a humidified atmosphere, and subcultured at 80% confluency using 0.25% trypsin. Transmission electron microscopy (TEM) micrographs were obtained on a JEOL 1400 Flash microscope equipped with a lanthanum hexaboride “Lab Six” emitter and operating at a maximum accelerating voltage of 120 kV. A Malvern Zetasizer Nano ZS instrument provided the hydrodynamic diameter and zeta potential measurements. Flow cytometry was performed on a Cytek Aurora spectral cytometer. The magnetic properties of the iron oxide nanoparticles were assessed on a superconducting quantum interference device (SQUID) from Quantum Design (Model MPMS3). The sample was placed inside a plastic bag (10 mm × 15 mm), which was then inserted into a plastic straw (11 cm long), and measurements were recorded at 100 K. Metal ion concentrations were quantified on an Inductively Coupled Plasma-Optical Emission Spectroscopy (ICP-OES) apparatus from Varian (Model Varian 710). Optical density was measured on a Molecular Devices plate reader (Model SpectraMax M3). A Nikon Eclipse TS2R inverted light microscope was used to collect images of Prussian blue stained cells.

### 2.2 Synthesis of the G2HA polymer

The G2HA polymer was synthesized following the Ugi reaction as described by Liu and colleagues.^26^ In brief, 3-phenylpropyl amine (35 µL in 200 µL of ethanol) was mixed with an aqueous solution of HA (100 mg), and the pH was adjusted to 4 using hydrochloric acid (1 M). Formaldehyde (3.5% in ethanol, 100 µL) and cyclohexyl isocyanide (28 µL in 172 µL ethanol) were added to the mixture, which was then stirred for 1 hour at room temperature. The pH was subsequently increased to 12 by adding an aqueous solution of sodium carbonate (2 M) to hydrolyze the ester byproduct, and stirring was continued for 2 days. The mixture was dialyzed against water using a dialysis tube with a MWCO of 3500 Da, and freeze-dried to obtain G2HA as a white solid. The degree of functionalization was 28% per disaccharide as determined by ^1^H-NMR.

### 2.3 Synthesis of iron oxide nanoparticles

The thermal decomposition method was employed to synthesize the oleic acid-coated iron oxide nanoparticles (OA-IONPs) as described by Sun and coworkers.^27^ In brief, a mixture of Fe(acac)_3_, oleic acid, oleylamine, 1,2-hexadecanediol (1 : 3 : 3 : 5 molar ratio, respectively) in benzyl ether was flushed with nitrogen gas at room temperature for 1 hour. Nucleation of the nanoparticles was achieved by heating the reaction mixture to 200 °C for 2 hours. Subsequent heating of the mixture to 320 °C for 1 hour ensured the growth of the iron oxide nanoparticles. The synthesized Fe_3_O_4_ nanoparticles were first cooled to ambient temperature, followed by the addition of ethanol, and isolated *via* magnetic separation. The nanoparticles were washed with ethanol (3 times) and then dried in an oven set at 100 °C for 1 hour. The black powder (0.4 g) was collected and stored at room temperature for further analysis and use.

### 2.4 Synthesis of HA-IONPs and G2HA-IONPs

The biphasic ligand exchange method was utilized to coat the iron oxide nanoparticles with HA or G2HA.^28^ The hydrophobic OA-IONPs (25 mg) were dissolved in 15 mL toluene, while HA or G2HA (50 mg) was dissolved in deionized water containing 0.5 mL of 0.5 N potassium hydroxide (KOH). The biphasic mixture was heated between 120 and 130 °C for 24 hours with vigorous stirring, after which it was cooled to room temperature and stirred for additional 6 hours. The aqueous layer containing HA-capped IONPs was collected from a separatory funnel. Excess HA and KOH were removed using Amicon® Ultra-15 Centrifugal Filter Units at a speed of 4000 rpm. The concentrated nanoparticle solution was diluted to 20 mL and placed on an external magnet to remove large aggregates. The amounts of iron in HA-IONPs and G2HA-IONPs were then standardized using ICP-OES. The concentration of iron in either sample was extrapolated from a standard curve generated from five iron solutions prepared from standard iron solution (1000 ppm).

### 2.5 Synthesis of FITC-G2HA-IONPs

The FITC-labelled G2HA-IONPs were prepared according to the procedure described in a previous report.^28^ In brief, fluorescein isothiocyanate (FITC) (0.1 g, 0.26 mmol) was reacted with excess adipic dihydrazide (ADH) (0.25 g, 1.45 mmol) in 30 mL of bicarbonate/carbonate buffer (pH 9) for 1 hour at room temperature in the dark. Adjusting the pH of the reaction mixture to 3.5 through the dropwise addition of 0.1 N HCl solution caused the precipitation of the desired FITC-ADH conjugate, which was washed with water and dried by lyophilization. FITC-ADH was conjugated onto HA-IONPs or G2HA-IONPs using EDCI coupling chemistry. In brief, 10 mg of the nanoparticles were dissolved in 20 mL of deionized water, and the pH was adjusted to 4.5 with 0.1 N HCl solution. EDCI (1 mg) dissolved in H_2_O was added, and the carboxyl groups were activated for 1 hour. FITC-ADH (5 mg) was added next, and the reaction mixture was stirred for 6 hours at room temperature, after which it was purified by dialysis (MWCO of 3500) and using Amicon® Ultra-15 Centrifugal Filter Units (MWCO of 100 000).

### 2.6 Assessing the suitability of G2HA-IONPs for MPI

The suitability of the synthesized nanoparticles as an MPI tracer material was evaluated by imaging phantoms composed of serial dilutions of the hyaluronan-coated IONPs (50 µL in 1.5 mL Eppendorf tubes) on a MOMENTUM MPI scanner from Magnetic Insight Inc. The tubes were taped into a sample holder, and the optimal MPI operating parameters for obtaining images with high resolution and sensitivity were identified as follows: scan type: 2D scan; scan mode: standard; Z FOV: 5.0 cm; 5.7 T m^−1^ gradient. MPI signals were subsequently extrapolated using VivoQuant's 3D ROI tool.

### 2.7 Cellular uptake of HA-IONPs and G2HA-IONPs by 4T1 breast cancer cells

#### 2.7.1 Cellular localization of nanoparticles by TEM

4T1 cancer cells were cultured in a 10 cm Petri dish at 5 × 10^6^ cells per plate and incubated overnight at 37 °C and 5% CO_2_. Growth medium was removed, G2HA-IONPs were added at a working iron concentration of 10 µg mL^−1^ (in RPMI 1640), and the plate was incubated for 6 hours. The nanoparticle solution was then removed, the cells were washed with PBS (three times), and trypsin (1 mL) was added to detach the cells for 5 min. FBS-containing RPMI 1640 (10 mL) was added, and the cells were transferred to 15 mL centrifuge tubes. The cells were pelleted by centrifugation and washed successively with PBS. The cells were then transferred to a 1.5 mL Eppendorf tube, and the fixing reagent (0.5 mL), comprising paraformaldehyde/glutaraldehyde (2.5% each) in 0.1 sodium cacodylate buffer (pH 7.4), was added and incubated with the cells for 12 hours at 4 °C. This was followed by pelleting the cells by centrifugation (2500 rpm), embedding the pellet in 2% agarose, treating it with 1% OsO_4_ in 0.1 M sodium cacodylate buffer, and dehydrating it by serial dilutions of acetone. Finally, poly/bed 812 resin was used to embed the dehydrated cells, and sections were placed on TEM copper grids. The cells were imaged by TEM.

#### 2.7.2 Quantitative uptake of nanoparticles by Prussian blue staining assay

4T1 breast cancer cells were cultured in a 24-well plate (3 × 10^4^ cells per well) in FBS-containing RPMI 1640 for 24 hours at 37 °C and 5% CO_2_, after which the growth medium was removed and cells were washed with PBS three times. HA-IONPs or G2HA-IONPs were added to the cells at an iron concentration of 10 µg mL^−1^ (in RPMI 1640), and cells were incubated for 6 hours. The nanoparticles' solution was then removed, and 4T1 cells were washed with PBS, treated with trypsin, collected by centrifugation in 1.5 mL Eppendorf tubes. 6 N HCl solution (750 µL) was added to the pelleted cells and the suspension was agitated for 2 hours to induce the lysing of the cells. The mixture was then centrifuged (10 000 rpm) to remove the debris, and the supernatant was transferred to 1.5 mL Eppendorf tubes. 4% K_4_FeCN_6_ aqueous solution (750 µL) was added next, and the mixture was incubated in the dark at room temperature for 10 min to allow the development of blue color. Aliquots (100 µL) were transferred to a 96-well plate, and optical density (OD) was measured on an ELISA plate reader at 700 nm.

For competition experiments, 4T1 cells (5 × 10^4^ cells per well) in a 6-well plate were first incubated with free HA (10 mg mL^−1^, 1 mL) for 2 hours, followed by adding G2HA-IONPs in 1 mL of RPMI 1640 (Iron working concentration = 12.5 µg mL^−1^). Non-competed cells without pre-incubation with free HA received the G2HA-IONPs solution at the same iron working solution. Cells were incubated at 37 °C and 5% CO_2_ for 5 hours, after which they were processed for analysis by the quantitative Prussian blue assay as described above.

For assessing the energy dependent uptake of G2HA-IONPs, 4T1 cells (5 × 10^4^ cells per plate) were cultured in 35 mm plates at 37 °C and 5% CO_2_. After removing the culture medium and washing the cells with PBS, the plates received G2HA-IONPs in 1 mL of RPMI 1640 at a working iron concentration of 12.5 µg mL^−1^. Some plates were incubated at 37 °C while others at 4 °C in a refrigerator for 3 hours. Control plates received FBS-free RPMI 1640 medium and maintained at 37 °C. Following incubation, the cells were collected and the amount of iron taken up by cells was determined by the quantitative Prussian blue assay as described above.

#### 2.7.3 Quantitative uptake of nanoparticles by ICP-OES

4T1 cancer cells (5 × 10^4^ cells per well) were adhered to the bottom of a 6-well plate. Following the aspiration of the growth medium, each well received 2 mL of HA-IONPs or G2HA-IONPs at a working concentration of iron equivalent to 10 µg mL^−1^, and the plate was incubated at 37 °C for 6 hours. The nanoparticle solutions were then removed, the wells were washed with PBS, and the cells were collected by trypsinization. The cells were transferred to 1.5 mL Eppendorf tubes by centrifugation at 4 °C and washed with PBS. This process was repeated 3 times. The collected cells were digested with 70% HNO_3_ (0.5 mL) at 70 °C for 3 hours, after which the cellular debris was removed by centrifugation. The collected supernatant was diluted with water to attain 2% HNO_3_ solution suitable for analysis by ICP-OES. The concentration of iron taken up by the cells was extrapolated from a standard curve generated from five known concentrations of iron. A similar approach was used to evaluate the CD44-mediated cellular uptake of G2HA-coated nanoparticles. Briefly, 4T1 (CD44^+^) and MCF-7 cells (CD44^−^) were incubated with the nanoparticles at a working concentration of 12.5 µg Fe per mL, and the intracellular iron content was subsequently quantified by ICP-OES.

#### 2.7.4 Uptake of FITC-G2HA-IONPs by laser confocal microscopy

4T1 cancer cells (1 × 10^4^ cells per well) were adhered to the bottom of a 4-well chambered plate suitable for confocal microscopy. The culture medium was removed, FITC-G2HA-IONPs in RPMI 1640 were added at an iron working concentration of 10 µg mL^−1^ (0.5 mL), and the cells were incubated for 5 hours. Negative control cells received RPMI 1640 only (0.5 mL). Lysotracker red DND-99 (1 µM; 50 µL per well) was added one hour before the end of incubation. The supernatant was removed and the cells were washed twice with PBS. The cells were fixed using 10% formalin (250 µL per well) for 15 min, after which the fixing solution was removed, and the cells were washed twice with PBS. DAPI (300 nM, 200 µL per well) was added, and the cells were incubated for 5 minutes. DAPI was removed, and the cells were washed with PBS, followed by water. The plate was stored in the dark at 4 °C until analysis time on a Leica Stellaris 5 White Light laser confocal microscope. A similar experimental protocol was followed to evaluate the uptake of the nanoparticles by MCF-7 cancer cells cultured in DMEM.

#### 2.7.5 Effect of β-cyclodextrin on the uptake of FITC-G2HA-IONPs by flow cytometry

4T1 cancer cells (5 × 10^4^ cells per well) were cultured in a 24-well plate overnight at 37 °C and 5% CO_2_. The supernatant was aspirated, and the cells were washed with PBS. Some of the wells received 0.5 mL of β-cyclodextrin in RPMI 1640 at a working concentration of 5 mM, while the other wells received RPMI 1640 only.^29^ The plate was incubated at 37 °C for 30 minutes, after which FITC-G2HA-IONPs or G2HA-IONPs were added to the test wells at an iron working concentration of 5 µg mL^−1^, while negative control wells received RPMI 1640. The plate was incubated for additional 2 hours after which the supernatant was removed, and the cells were washed with PBS. The cells were collected by adding trypsin (0.2 mL), which was neutralized afterwards with FBS-containing RPMI 1640 (2 mL). The cells were pelleted by centrifugation and washed with PBS three times, fixed with 10% formalin, and transferred in PBS to FACS tubes. FITC fluorescence was monitored on an Aurora flow cytometer.

### 2.8 MTS cell viability assay

4T1 cancer cells (5 × 10^3^ cells per well) were cultured in a 96-well plate overnight at 37 °C and 5% CO_2_. The culture medium was removed, and G2HA-IONPs in RPMI 1640 were added to the test wells at different iron concentrations (0.00128–100 µg mL^−1^). Control wells received RPMI 1640 only. The plate was incubated at 37 °C for 6 hours, after which the nanoparticles were removed and the cells were washed with PBS. FBS-containing RPMI 1640 was added to all wells (200 µL per well), and the plate was incubated for 18 hours at 37 °C. The MTS reagent was added (20 µL per well), and the plate was incubated in the dark for 3 hours at 37 °C. Optical density at 490 nm was then measured on an ELISA plate reader. The percentage of viable cells was determined relative to control wells that did not receive nanoparticle treatment.

### 2.9 Imaging of 4T1 cells by MPI

4T1 cells (2 × 10^5^ cells per well) immobilized in a 6-wellplate received HA-IONPs or G2HA-IONPs in RPMI 1640 at the same iron concentration (10 µg mL^−1^) and incubated at 37 °C and 5% CO_2_ for 6 hours. Control cells received RPMI 1640 only. The treatment solution was then removed, and the cells were washed with PBS. Trypsin was added (300 µL per well), and the detached cells were treated with FBS-containing RPMI 1640 (5 mL per well). The cells were collected by centrifugation (2500 rpm, 4 °C), transferred to 1.5 mL Eppendorf tubes, and pelleted by centrifugation. The supernatant was removed, and the cells were fixed in 10% formalin for 5 min, washed with PBS, and pelleted. 1% agarose solution, prepared by microwave heating of agarose in deionized water, was added to the tubes (0.3 mL per tube) and allowed to cool to room temperature. The prepared samples were stored at 4 °C until imaging on the MPI scanner. MPI images were obtained on a MOMENTUM MPI scanner from Magnetic Insight Inc. Tubes were taped into a sample holder. The parameters applied for imaging were as follows: scan type: 2D scan; scan mode: standard; Z FOV: 5.0 cm; 5.7 T m^−1^ gradient. MPI signals were subsequently extrapolated using VivoQuant's 3D ROI tool.

### 2.10 Statistical analysis

The assumption of normality was met and confirmed with the Shapiro–Wilk test (*p* > 0.05), validating the use of Tukey's HSD test for *post-hoc* analysis. Tukey's HSD was used to analyze pairwise differences between treatment groups following a significant ANOVA result. Most comparisons showed statistically significant differences, confirming that treatment effects differ across groups. Differences in the mean values among the treatment groups are considered statistically significant at *p* ≤ 0.001.

## 3 Results and discussion

### 3.1 Synthesis of the G2HA derivative

**Scheme 1:**
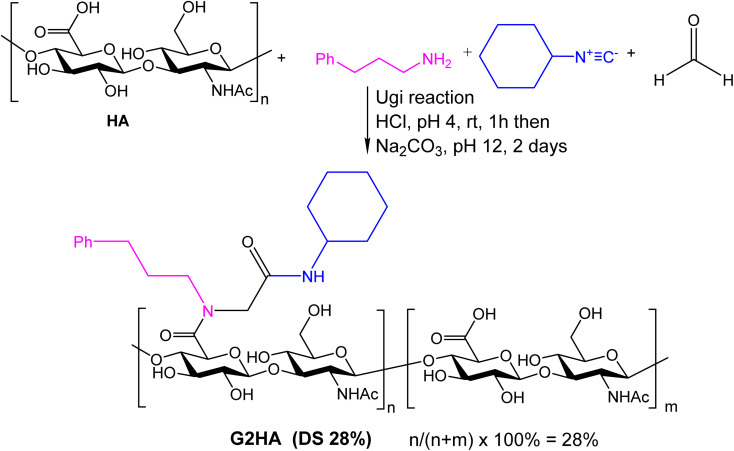
Synthesis of the modified hyaluronan analogue G2HA under the Ugi reaction conditions. The reactants were reacted at pH 4 for 1 hour, after which the pH was increased to 12 to hydrolyze the ester byproduct resulting from the Passerini reaction.

The G2HA analogue was synthesized *via* the Ugi reaction by reacting hyaluronan (31 kDa), 3-phenylpropyl amine, cyclohexyl isocyanide, and formaldehyde at pH 4.0 as represented in Scheme 1. The success of the reaction and the degree of the substitution were validated by NMR analysis (Fig. S1). The appearance of aromatic protons in the ^1^H-NMR spectrum and three different carbonyl groups in the ^13^C-NMR spectrum of G2HA supported the successful functionalization. These peaks were absent in the spectra of unmodified hyaluronan. Integrating the aromatic protons in the ^1^H-NMR spectrum of G2HA relative to the anomeric protons of the disaccharide unit suggested that the degree of substitution (DS) is 28%.

### 3.2 Preparation of IONPs

**Fig. 1:**
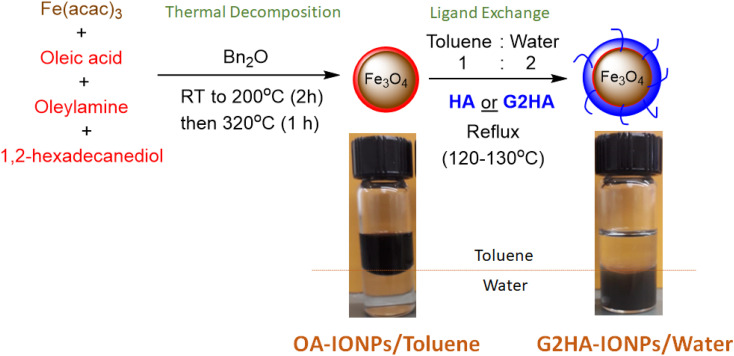
Preparation of the OA-IONPs by the thermal decomposition method and G2HA-IONPs by the ligand exchange method.

The IONP core was prepared by the thermal decomposition method, which is well known for the assembly of monodisperse, uniformly sized (narrow size distribution), crystalline, spherical nanoparticles.^27^ The experimental conditions summarizing the applied protocol are depicted in Fig. 1. The resulting oleic acid (OA)-coated IONPs (OA-IONPs) are hydrophobic in nature and therefore are well dispersed in non-polar solvents such as toluene. The unmodified HA polymer or G2HA was anchored onto the iron oxide nanoparticle surface by the ligand exchange method, where a biphasic system composed of an aqueous solution of the hyaluronan adduct and a toluene solution containing the OA-IONPs was refluxed between 120 and 130 °C.^28^ Functional groups like carboxyl and hydroxyl groups of HA can efficiently displace OA from IONPs, thus shifting their solubility from toluene to water (Fig. 1). Unlike other methods, utilizing the ligand exchange method to attach HA analogues on the surface of the nanoparticles requires no prior functionalization of the biopolymer, thus helping to maintain optimal affinity of HA to its receptors such as CD44.

**Fig. 2:**
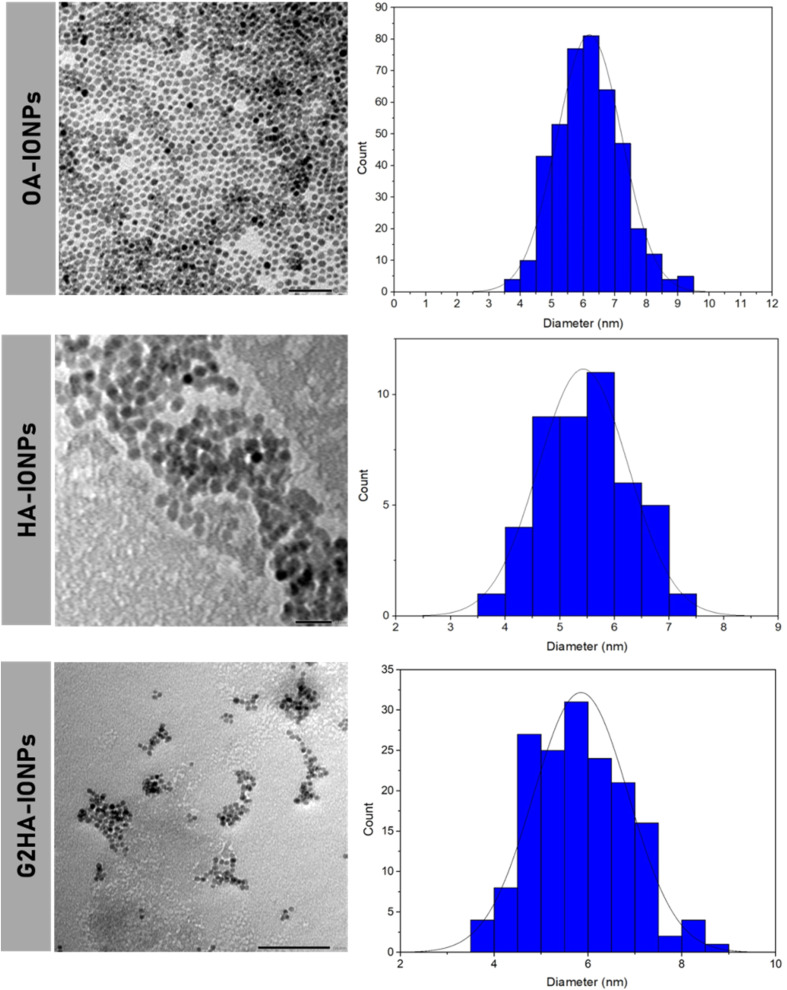
TEM images of OA-IONPs, HA-IONPs, and G2HA-IONPs and the corresponding size distribution histograms (scales: 50 nm, 20 nm, and 100 nm, respectively).

TEM micrographs revealed the high monodispersity and size uniformity of the nanoparticles with an inorganic metal core of 5.7 ± 2.0 nm in size (Fig. 2). The morphology and size of the inorganic core did not change following the incorporation of the HA analogues on IONPs, highlighting the suitability of the exchange method for preparing surface-modified nanocomposites.

The hydrodynamic diameter of the various nanoformulations was determined by dynamic light scattering (DLS), revealing sizes of 113.53 ± 0.49 nm and 135.03 ± 1.53 nm for HA-IONPs and G2HA-IONPs, respectively (Fig. S2). Importantly, the polydispersity index of all nano-preparations ranged between 0.164 and 0.175 (below 0.4), highlighting the applicability of the utilized synthetic method for the preparation of close-to-monodisperse nanoparticles with minimal aggregation, thus corroborating the findings of the TEM images. The disparity in the nanoparticle size determined by TEM and DLS is due to the fact that TEM measures the size of the dried iron oxide nanoparticle core. However, DLS measures the larger effective size of the nanoparticle in solution, which includes the particle core, its surface coating, and the surrounding solvent shell.

All nanoformulations exhibited a highly negative zeta potential with values of −27.7 ± 5.3 mV and −44.2 ± 7.5 mV for HA-IONPs and G2HA-IONPs, respectively. Such negative values minimized aggregation of the nanoparticles and enhanced their dispersion in solution (Table 1 and Fig. S2). Monodispersity of nanoparticles is highly desired in biomedical applications such as MPI, ensuring reproducible and predictable biological profile, optimized cellular uptake, and consistent high-resolution imaging.

**Table 1:** Summary of the iron core size, hydrodynamic diameter, size distribution (polydispersity index, PDI), and surface charge (zeta potential) of the various IONPs

Nanoparticles	IONP core diameters (nm) (from TEM)	Hydrodynamic diameter (nm)	Polydispersity index	Zeta potential (mV)
OA-IONPs	5.7 ± 2.0	—	—	—
HA-IONPs	5.5 ± 1.8	113.53 ± 0.49	0.170 ± 0.008	−27.7 ± 5.3
G2HA-IONPs	5.5 ± 2.3	135.03 ± 1.53	0.185 ± 0.012	−44.2 ± 7.5

### 3.3 Assessing the magnetic properties of the nanoparticles

**Fig. 3:**
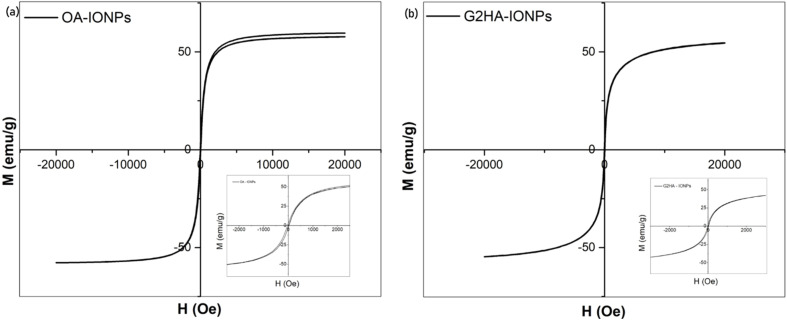
Magnetic hysteresis curves of (a) OA-IONPs and (b) G2HA-IONPs showing the superparamagnetic properties of the synthesized magnetic nanoparticles. Magnetization measurements were recorded at 100 K and are expressed as electromagnetic units per gram of IONPs (emu g^−1^). The insets in the figures show a magnified view of the region from −2000 to +2000 Oe on the *x*-axis to highlight low magnetic remanence (*M*_r_) and coercivity (*H*_c_).

A major goal of the current study is to prepare targeted IONPs that are efficient contrast agents for MPI. As such, it is essential to assess the magnetic properties of synthesized polysaccharide coated nanoparticles to justify their utility as tracers in MPI. A SQUID magnetometer was used to extrapolate the necessary magnetic parameters including magnetic saturation (*M*_s_), magnetic remanence (*M*_r_), and coercivity (*H*_c_), and their values are summarized in Table 2. OA-IONPs exhibited an *M*_s_ value of 56.2 emu g^−1^ (Fig. 3). The ligand exchange method utilized to anchor the G2HA polymer onto the surface of IONPs marginally affected the magnetization saturation, and G2HA-IONPs recorded an *M*_s_ value of 54 emu g^−1^. Such high *M*_s_ values reflected the potential of the nanoparticles in various applications such as magnetic separation, magnetically targeted delivery, improved data storage density, and efficient hyperthermia therapy.^30–32^ Specifically, high magnetization saturation *M*_s_ is an essential attribute of MPI contrast agents, providing optimal contrast, sharper spatial resolution, and higher sensitivity and enabling linear quantitative imaging.^33^ In addition, MPI contrast agents with high *M*_s_ values are characterized by minimal relaxation losses, thus maintaining the fidelity of the harmonic signal that is crucial for MPI.^34^ Contrast agents with high *M*_s_ values generate considerable MPI signals, enabling the usage of low doses for imaging and reducing operational cost and the concerns of potential toxicities *in vivo*. Another important parameter for effective MPI contrast agents is magnetic remanence *M*_r_, which reflects the ability of a material to retain magnetization after the removal of an applied external magnetic field. The *M*_r_ values of IONPs prepared in the current study are close to zero (2.65 emu g^−1^ for OA-IONPs and 2.92 emu g^−1^ for G2HA-IONPs), indicating that the magnetic characteristics of the nanoparticles are readily controllable (easily magnetized and demagnetized).^35^ Magnetic nanoparticles with low *M*_r_ are highly desirable in biomedical applications. The fact that the prepared nanoparticles do not retain much residual magnetization (low *M*_r_) prevents aggregation of the nanoparticles, enhances colloidal stability in solutions, and reduces potential toxicity.^36^ Such an attribute is essential for improved sensitivity and the generation of clean MPI signals when smaller amounts of the contrast agent are used. In addition to *M*_r_, coercivity (*H*_c_) was also determined, which is defined as the external magnetic field required to bring *M* to zero following magnetization of the material to saturation. *M*_r_ and *H*_c_ values close to zero are characteristic of superparamagnetic materials that can act as ideal MPI tracers, which fits the magnetic profile of the IONPs prepared in this study (Fig. 3). This is further supported by the low squareness ratio (*M*_r_/*M*_s_) for OA-IONPs (0.044) and G2HA-IONPs (0.053), which is consistent with the narrow hysteresis loops and reflecting smaller hysteresis losses. These characteristics are typically important for the application of magnetic nanoparticles in magnetic imaging, which requires feasible and spontaneous relaxation of magnetization without the retention of magnetic memory.

**Table 2:** Magnetic parameters of the prepared nanoparticles. Magnetization measurements were recorded at 100 K

Nanoparticles	Saturation magnetization (*M*_s_)	Magnetic remanence (*M*_r_)	Coercivity (*H*_c_)	Squareness ratio (*M*_r_/*M*_s_)
OA-IONPs	59.6 emu g^−1^	2.65 emu g^−1^	15.5 Oe	0.044
G2HA-IONPs	54.6 emu g^−1^	2.92 emu g^−1^	15.1 Oe	0.053

### 3.4 Hyaluronan-coated IONPs as MPI tracers

**Fig. 4:**
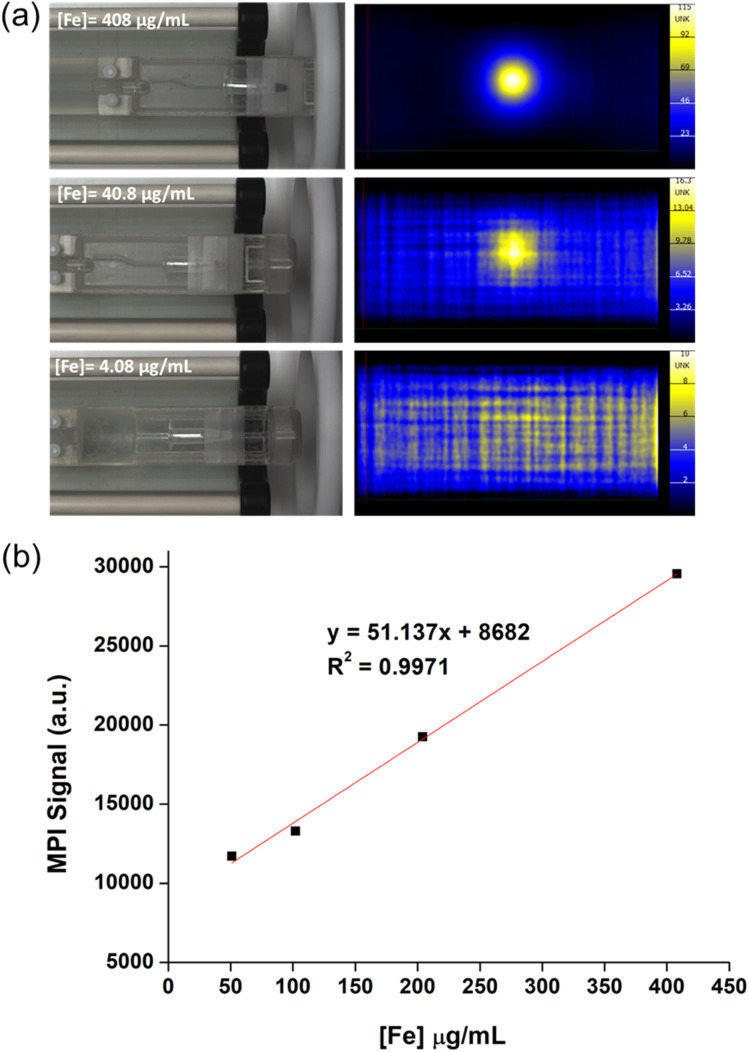
(a) Images showing the sample holder of the MPI scanner with attached Eppendorf tubes containing G2HA-IONP solutions at different concentrations, and the corresponding MPI signal; (b) plot depicting the linear dose-dependent MPI signal as a function of nanoparticle concentrations.

The desirable magnetic properties of the G2HA-coated IONPs enabled them to act as efficient MPI tracers. The suitability of the nanoparticles as MPI contrast agents and the linearity of the MPI signal at different concentrations of the nanoparticle solution were assessed, and the results are depicted in Fig. 4 for G2HA-IONPs and Fig. S3 for HA-IONPs. Both nanoparticle formulations generated a linear dose-dependent signal on the MPI scanner, with coefficients of determination (*R*^2^) close to unity (0.9971 for G2HA-IONPs and 0.9999 for HA-IONPs). Such an observation highlighted the suitability of the method to determine the concentration of iron from the generated MPI calibration curve when desired. The observed signal linearity is consistent with the high magnetization saturation *M*_s_ of the nanoparticles enabling quantitative imaging.^37^

### 3.5 Biocompatibility of G2HA-IONPs

**Fig. 5:**
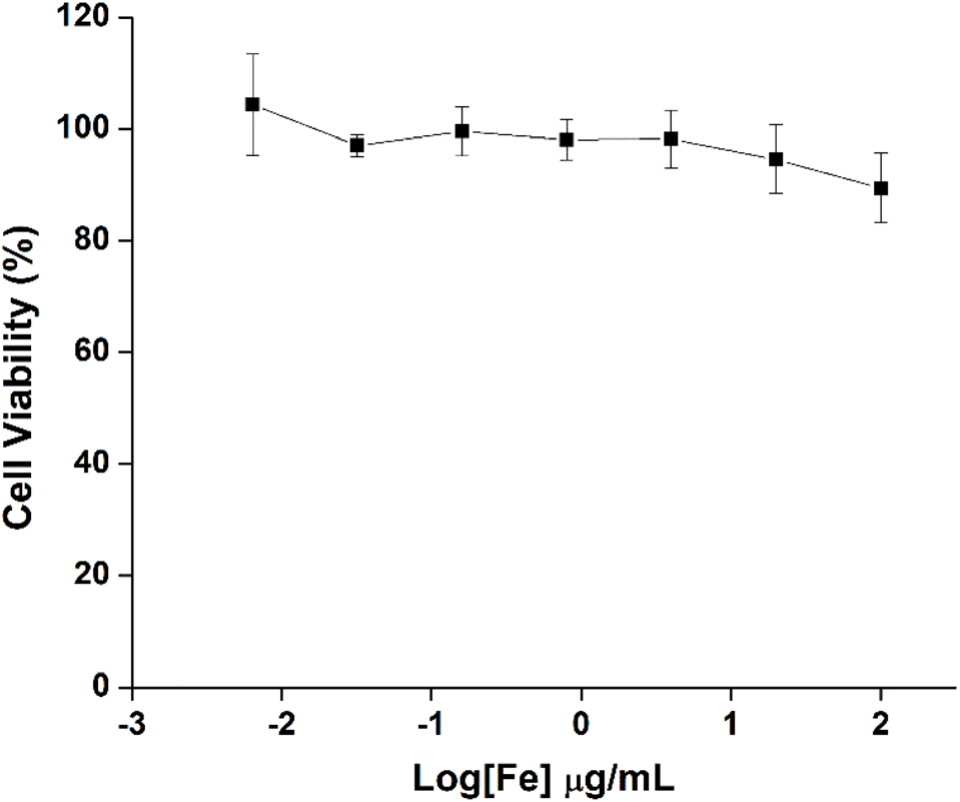
MTS cell viability assay of 4T1 breast cancer cells treated with different concentrations of G2HA-IONPs.

It is foundational to assess the biocompatibility of the iron oxide nanoparticles before using them in biomedical contexts to ensure the safety of the nanoformulations, define the safe dose range, and avoid misinterpretation of experimental results.^38^ In the current study, the biocompatibility of G2HA-IONPs was evaluated using the MTS cell viability assay, and the results are presented in Fig. 5. The tested nanoparticles demonstrated high biocompatibility, with over 90% viability of treated cells even at the highest concentration tested (100 µg Fe per mL).

### 3.6 Uptake of HA-IONPs and G2HA-IONPs by 4T1 cancer cells

**Fig. 6:**
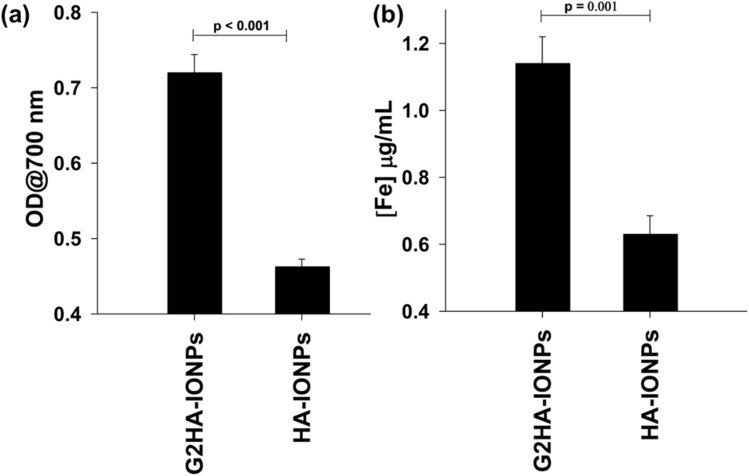
Quantification of the uptake of G2HA-IONPs and HA-IONPs by 4T1 cancer cells using (a) the Prussian blue staining assay and (b) ICP-OES.

It has been shown that G2HA demonstrated significant enhancements in binding to CD44 compared to the native HA.^26^ The expression of CD44 by the 4T1 cell line used in this study has been validated in our previous report.^15^ The enhanced binding affinity translated into increased uptake of G2HA by CD44-overexpressing cancer cells. In this report, G2HA was anchored onto IONPs, and its ability to increase the uptake of the nanoparticles compared to HA was assessed in 4T1 breast cancer cells, which are known to overexpress the CD44 receptor.^15,39^ The cells were incubated with G2HA-IONPs or HA-IONPs at 10 µg Fe per mL for 6 hours. The internalization of the nanoparticles was validated visually by staining the iron oxide core with potassium ferrocyanide to produce the characteristic Prussian blue stain. Quantitation of the internalized nanoparticles was achieved by monitoring the absorbance of the Prussian blue stain at its characteristic wavelength (700 nm) on a UV-vis spectrophotometer, and the obtained results are presented in Fig. 6a. Furthermore, the intracellular iron content was quantified by ICP-OES as revealed in Fig. 6b. The obtained results are presented in Fig. 6a and b, respectively. Interestingly, G2HA-IONPs showed remarkable uptake by the 4T1 cancer cells, demonstrating statistically significant enhancement over HA-IONPs as revealed by UV-vis spectrophotometry and ICP-OES, respectively. This implied that anchoring G2HA onto IONPs did not alter its superior binding profile to CD44 compared to HA. The enhancement in the relative uptake of nanoparticle formulations can further be intensified by avidity, defined as the simultaneous interaction between multiple ligands on the nanoparticle with cell surface receptors, resulting in very strong overall binding even if affinity is moderate.^40^ It has been validated that stronger binding affinities can contribute to higher avidity.^41^ Multivalent binding interactions trigger receptor clustering and effectively activate receptor-mediated endocytosis particularly in tissues that express high densities of the target receptor such as the cancerous tissue.^42^ Therefore, the stronger binding of G2HA with CD44 compared to that by HA, particularly in a nanoparticle formulation, can induce higher avidity and improved uptake by CD44-overexpressing cells.

### 3.7 Internalization of the G2HA-IONPs: mechanistic insights into receptor-mediated endocytosis

**Fig. 7:**
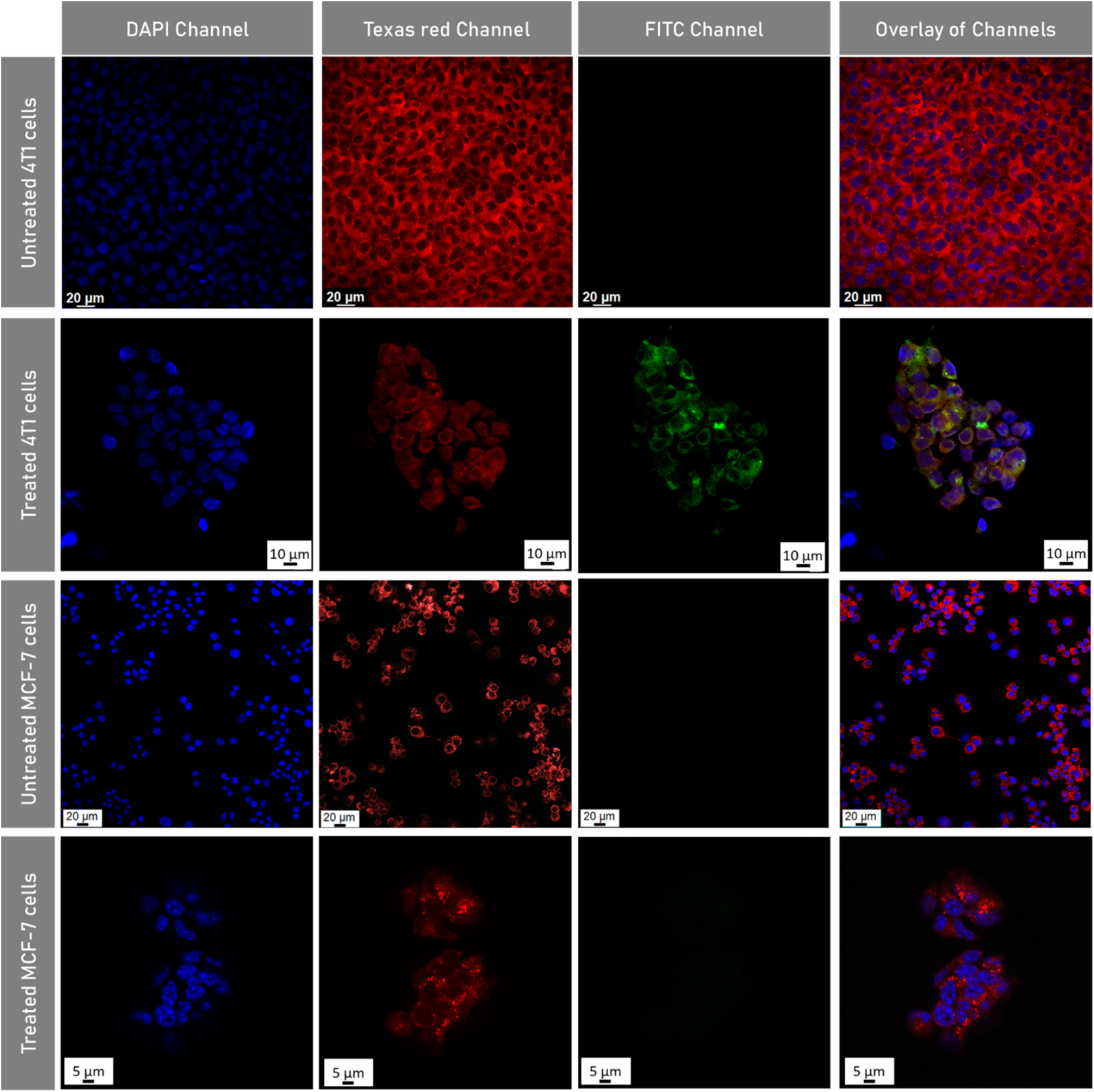
Laser confocal microscopy images for breast cancer cells that received FITC-labeled G2HA-IONPs (treated 4T1 cells and treated MCF-7 cells) or culture medium (RPMI 1640 for untreated 4T1 cells or DMEM for untreated MCF-7 cells). DAPI, LysoTracker Red, and FITC dyes were used to stain nuclei, lysosomes, and the G2HA-IONPs, respectively. The overlay channels show the merged images captured from the DAPI, Texas Red, and FITC channels.

The internalization of G2HA-IONPs by the 4T1 cancer cells and their intracellular localization were assessed by confocal microscopy, where laser confocal images were collected for 4T1 cancer cells treated with FITC-labelled G2HA-IONPs (Fig. 7). The FITC dye was covalently attached to G2HA *via* EDCI chemistry as shown in Scheme S1. LysoTracker Red dye was used to selectively stain lysosomes inside the cells and its fluorescence was registered on the Texas red channel. Simultaneously, the FITC dye on the nanoparticle was visualized on the FITC channel. No signal was collected on the FITC channel for the cells that received the growth media only without FITC-labeled nanoparticles. However, green fluorescence was registered in the FITC channel of the cells that received FITC-G2HA-IONPs, confirming that the nanoparticles were internalized by the cells. Interestingly, remarkable co-localization of the LysoTracker Red and FITC signals was recorded, producing purple color in the overlaid images. Such an observation validated that G2HA-IONPs were internalized into lysosomes inside the cells, and supported a receptor mediated endocytosis mechanism for the intracellular trafficking of the nanoprobes. Interestingly, the CD44-deficient MCF-7 breast cancer cells exhibited negligible fluorescence in the FITC channel under identical experimental conditions. The stark contrast in the uptake of the G2HA-IONPs by 4T1 and MCF-7 cells validated visually the targeting efficiency of the G2HA coating and ascertained that the cellular internalization of the targeted nanoparticles is highly dependent on the presence of the CD44 receptors that mediated the selective active targeting of cancer cells through receptor mediated endocytosis.

**Fig. 8:**
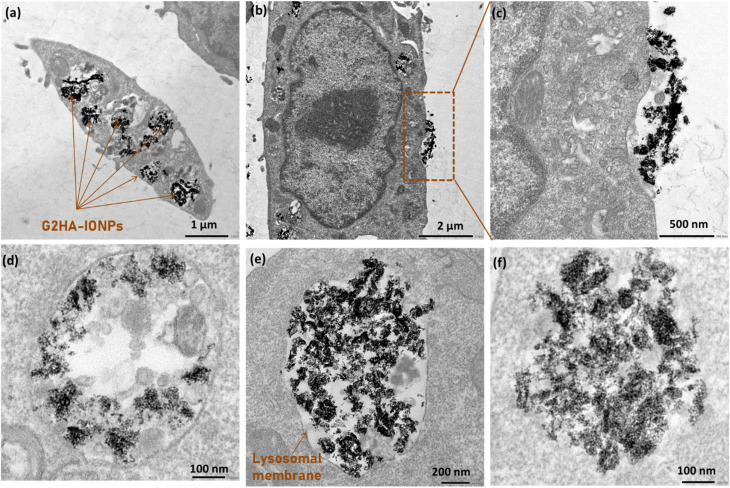
Visualization of the endocytosis of G2HA-IONPs. (a) Representative TEM image of a 4T1 cancer cell treated with G2HA-IONPs for 6 hours, showing the extensive uptake of nanoparticles by the cell; (b and c) attachment of the nanoparticles to the surface of the cell by binding to the CD44 receptors, forming a groove that engulfs the nanoparticles and pinches off to form an early endosome in the cytoplasm (d); (e) TEM micrograph showing the localization of the nanoparticles inside a lysosome; (f) disappearance of the lysosomal membrane and release of the nanoparticles into the cytoplasm.

TEM micrographs of the cells treated with G2HA-IONPs (Fig. 8) provided deeper mechanistic insights into the internalization of the nanoparticles by the cells. Fig. 8a revealed the extensive uptake of the G2HA-coated nanoparticles by cells, highlighting the efficiency of the synthesized nanoformulation to act as an effective MPI tracer capable of efficiently labelling, tracking, and imaging cells. The hyaluronan-coated nanoparticles are presumably internalized through CD44-mediated endocytosis. Following the interaction of the G2HA coating on the nanoparticles with the CD44 receptors on cells, the endocytosis process is initiated by the formation of a groove on the cell membrane (Fig. 8b and c). This groove could grow to encapsulate the HA–CD44 complex, and eventually pinch off into the cytoplasm forming an early endosome where nanoparticle clusters can be seen attached to the periphery of the formed vesicle (Fig. 8d). As the pH decreases in the endosome to become a lysosome, the carboxyl groups of the hyaluronan derivative may become protonated, which increases its hydrophobicity. As a result, the magnetic nanoparticles aggregate, leading to the formation of clusters that fill the lysosome (Fig. 8e). The nanoparticles appear to escape from the lysosome as evident from the disappearance of the lysosomal membrane that was encapsulating the nanoparticle cluster observed in some images (Fig. 8f). It is possible that the G2HA polymer acted as a proton sponge, soaking up protons from the acidic lysosomal compartment through its carboxyl groups and disrupting its acidity balance, leading to its collapse and the release of IONPs into the cytoplasm.^43,44^

**Fig. 9:**
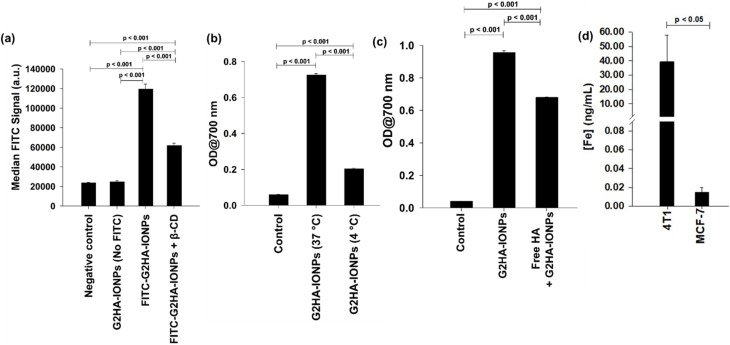
(a) Flow cytometry analysis of the uptake of FITC-G2HA-IONPs in the absence and presence of β-cyclodextrin (β-CD). The uptake of the nanoparticles dropped by 48% when β-cyclodextrin was added; (b) comparative study for the uptake of G2HA-IONPs by 4T1 breast cancer cells at 37 °C and 4 °C using the Prussian blue assay; (c) uptake of the G2HA-IONPs in the absence and presence of free hyaluronan; (d) cellular uptake of G2HA-coated nanoparticles by 4T1 (CD44-expressing) and MCF-7 (low-CD44 expressing) breast cancer cell lines following incubation for 6 hours and quantification of iron content by ICP-OES. Significantly higher iron content was observed in 4T1 cells compared to MCF-7 cells.

Several experiments were conducted to validate the proposed HA–CD44-mediated endocytosis mechanism. It has been reported that the endocytic CD44-mediated uptake of hyaluronan occurs through lipid-rich rafts and not clathrin-coated pits or caveolae.^45,46^ Therefore, adding a cholesterol solubilizer such as β-cyclodextrin would disrupt the cholesterol rich lipid rafts and adversely affect the CD44-mediated endocytosis of the nanoparticles. In fact, pre-incubation of the 4T1 cancer cells with β-cyclodextrin followed by the addition of the FITC-labeled G2HA-IONPs decreased the uptake of the nanoparticles by 48% as revealed by flow cytometry (Fig. 9a). Such an observation supported the endocytosis of G2HA-IONPs and asserted that the lipid-rich rafts played a pivotal role in the uptake mechanism.

A major characteristic of receptor-mediated endocytosis is that it is an energy dependent process.^47^ In other words, endocytosis will dramatically slow down at low temperatures (*e.g.* 4 °C). To further validate that endocytosis is the mechanism of internalization of G2HA-IONPs, the uptake of the nanoparticles was studied at 37 °C and 4 °C, and cellular uptake was analyzed by the Prussian blue assay. Fig. 9b summarizes the obtained results. Interestingly, 72% reduction in the uptake of G2HA-IONPs was observed when the experiments were performed at 4 °C relative to those conducted at 37 °C, suggesting that the IONP uptake process was energy dependent and supporting the endocytosis mechanism.

To confirm the role of the HA receptor in mediating the uptake of the G2HA-IONPs, the uptake of the nanoparticles was evaluated in the presence of excess free hyaluronan, and the amount of nanoparticles internalized by the 4T1 cancer cells was assessed by the Prussian blue assay. The uptake of the G2HA-IONPs was significantly reduced by 28% in the presence of free HA (Fig. 9c), suggesting that HA and G2HA-IONPs competed for the same binding sites on the cells and confirming the vital role played by G2HA in mediating the endocytosis of the nanoparticles. The HA competition approach employed in the current study provided supportive evidence for the CD44-mediated endocytosis mechanism by blocking receptors on the same cell type, thus eliminating the influence of cell line-to-cell line variabilities in terms of metabolic and growth rates, membrane structures, and non-specific endocytosis.^48–50^ The significant reduction observed in the HA-competition experiments validated the selectivity of G2HA-IONPs for CD44.

To further validate the CD44-mediated cellular internalization of the G2HA-coated nanoparticles, the uptake was comparatively evaluated in 4T1 (CD44^+^) and MCF-7 (CD44^−^) breast cancer cell lines. The intracellular iron content in both cell lines is presented in Fig. 9d. A striking difference in iron concentration was observed between the two cell lines, where the CD44-overexpressing 4T1 cells demonstrated substantial enhancement in intracellular iron (∼39 ng mL^−1^). However, the low CD44-expressing MCF-7 cells showed minimal uptake of the nanoparticles with iron content maintaining baseline values. The negligible uptake by the MCF-7 cells indicated that the non-specific internalization pathways contributed minimally to nanoparticle sequestration. This pronounced disparity in the uptake of the G2HA-coated nanoparticles supported the preferential accumulation in CD44-expressing cells and reinforced the role of the CD44 receptor in mediating the internalization of the nanoparticles. The observed uptake profile therefore validated the selectivity of the CD44-expressing 4T1 cells for the G2HA-functionalized nanoparticles with marginal off-target accumulation. The enhanced uptake in 4T1 cells is consistent with active targeting, which was likely prompted by the high affinity of the G2HA coating to the CD44 receptors triggering a receptor-mediated endocytic internalization of the nanoparticles. Overall, these findings underscored the role of the CD44 receptor as the primary gateway for the internalization of the G2HA-coated nanoparticles.

### 3.8 MPI imaging of 4T1 cancer cells

**Fig. 10:**
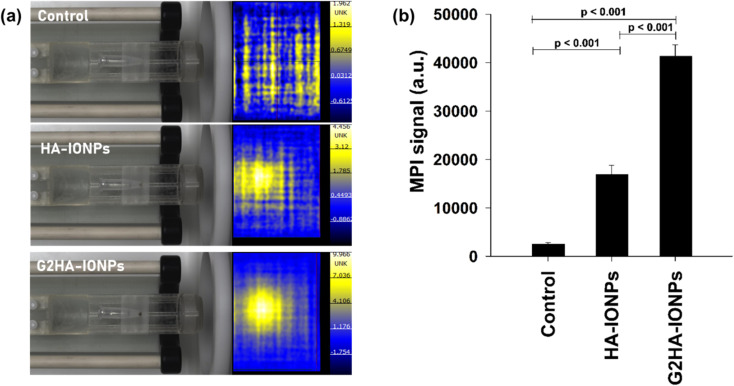
(a) Images showing the loading of the samples on the MPI sample holder and the generated MPI signals; (b) bar graph showing the quantification of the generated MPI signals.

Having confirmed that G2HA remarkably enhanced the uptake of IONPs compared to unmodified HA, it was crucial to validate that the enhanced uptake translated into sharper and more intense signals in MPI. Therefore, 4T1 breast cancer cells were incubated with HA-IONPs or G2HA-IONPs at identical iron concentration. The cells were washed to remove unbound particles and imaged on an MPI scanner as shown in Fig. 10. The control cells that did not receive any nanoparticle formulation produced a faint background MPI signal (Fig. 10a). However, cells that received G2HA-IONPs produced MPI signals 2.4 fold higher than those of the cells treated with HA-IONPs, highlighting that the enhanced uptake of G2HA-IONPs was concurrent with the improved MPI signal (Fig. 10b). Such an attribute is a testament to the suitability of the designed nanoparticles as contrast agents for MPI. The superior magnetic properties of the nanoparticles generated intense MPI signals with higher contrast and improved spatial resolution, enabling the accurate localization and quantification of the magnetic tracer. The magnetic profile of the prepared IONPs renders them reliable for various biomedical applications such as cell tracking, early detection, and monitoring the progression of diseases.

## 4 Conclusion

The current report described the anchoring of a novel hyaluronan derivative, termed G2HA and characterized by superior binding affinity to CD44 compared to the native HA, onto superparamagnetic iron oxide nanoparticles. The thermal decomposition method was used to prepare the IONP core, and the ligand exchange method was suitable for coating the nanoparticles with polysaccharides. The desirable magnetic properties of the prepared nanoformulations rendered them suitable targeted contrast agents for MPI. The enhanced binding affinity of G2HA to CD44 translated into prominent uptake by CD44-expressing cancer cells through receptor-mediated endocytosis and generated stronger dose-dependent signals on the MPI scanner. The unique characteristics of the prepared G2HA-IONPs support their use in efficient cell tracking, early disease detection, and longitudinal monitoring of diseases. While the results of the current study supported efficient and selective uptake of the targeted nanoparticles by cancer cells *in vitro*, achieving comparable selectivity *in vivo* is more complex, and further investigation is needed to validate their clinical applicability.

## Author contributions

Mohammad H. El-Dakdouki performed all experiments, conducted formal analysis, and wrote the original draft of the manuscript. Chia-wei Yang participated in MPI measurements, while A. K. M. Atique Ullah was involved in the preparation of the nanoparticles. Baraah U. Hijazi contributed to the formal analysis of the results. Fei Fan and Kunli Liu developed and assessed the binding affinity of the modified hyaluronan. Xuefei Huang led conceptualization and supervision of the project, and reviewed and edited the manuscript. All authors approved this version of the manuscript.

## Conflicts of interest

There are no conflicts to declare.

## Supplementary Material

NA-OLF-D5NA00979K-s001

## Data Availability

The data supporting the findings of this study are included in the article and its supplementary information (SI). Supplementary information is available. See DOI: https://doi.org/10.1039/d5na00979k.
