# Supplementary material for: Selective magnetic particle imaging of CD44 expressing cells using iron oxide nanoprobes functionalized with chemically modified hyaluronan
Source: Nanoscale Adv. 2026 Jun 16;8(15):4385–99. doi: 10.1039/d5na00979k (PMC13366091; doi:10.1039/d5na00979k)
Supplement: NA-008-D5NA00979K-s001 [file NA-008-D5NA00979K-s001.pdf]

Supplementary Information (SI) for Nanoscale Advances.
This journal is © The Royal Society of Chemistry 2026

Selective Magnetic Particle Imaging of CD44 Expressing Cells Using Iron Oxide Nanoprobes Functionalized with Chemically Modified Hyaluronan

Mohammad H. El-Dakdouki[a*], Chia-wei Yang[b], A. K. M. Atique Ullah[b], Fei Fan[b], Kunli Liu[b], Baraah U. Hijazi[b], and Xuefei Huang[b,c,d*]

[a] *Department of Chemistry, Faculty of Science, Beirut Arab University, P.O. Box 11-5020, Riad El Solh 11072809, Beirut, Lebanon*

[b] *Department of Chemistry, Michigan State University, 578 S. Shaw Lane, East Lansing, MI 48824, USA*

[c] *Institute for Quantitative Health Science and Engineering, Michigan State University, East Lansing, MI 48824, USA*

[d] *Department of Biomedical Engineering, Michigan State University, East Lansing, MI 48824, USA*

(SUPPORTING INFORMATION)

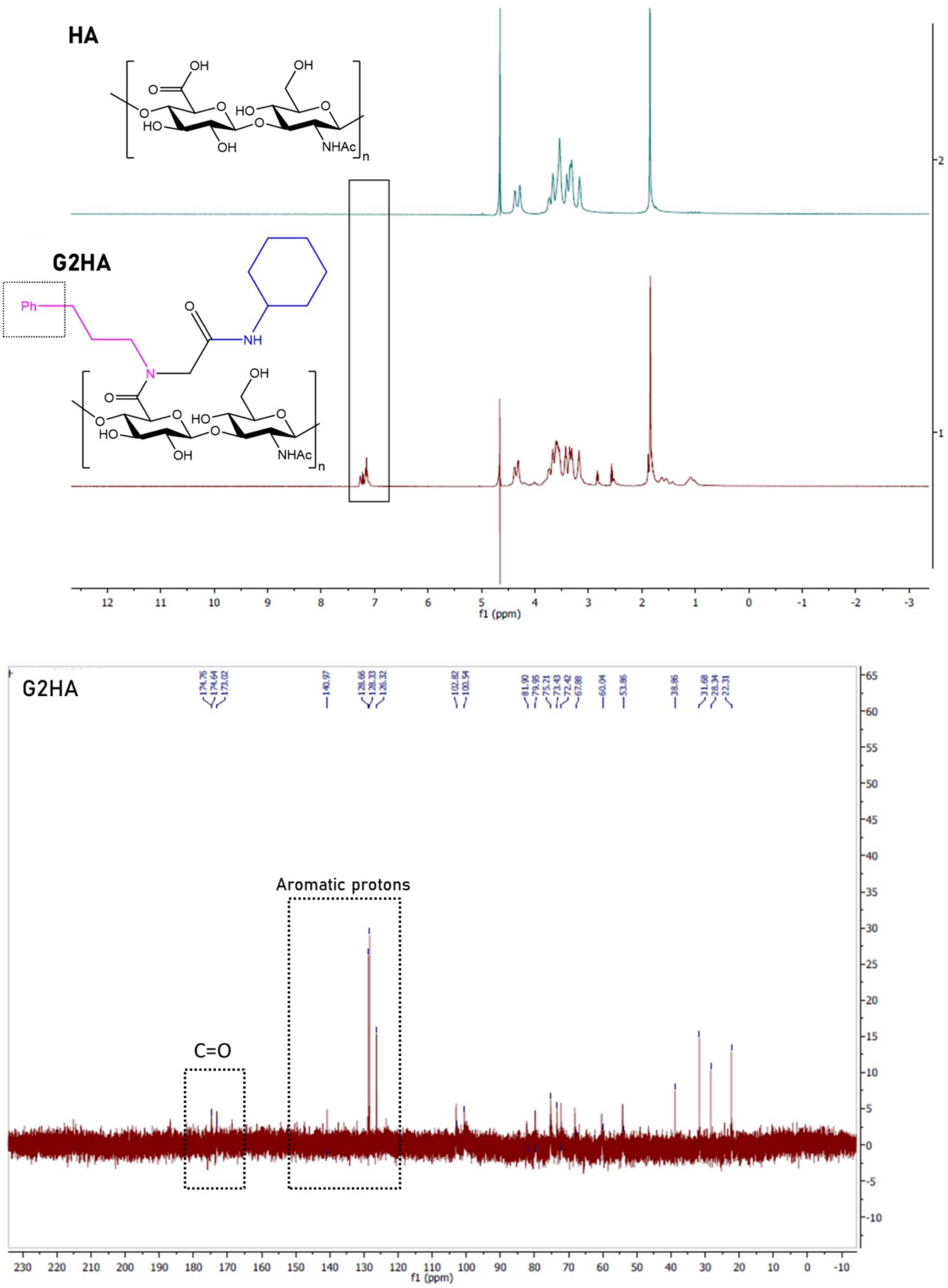

Fig. S1 (a) ^{1}H-NMR of HA and G2HA spectra; (b) ^{13}C-NMR spectrum for G2HA.

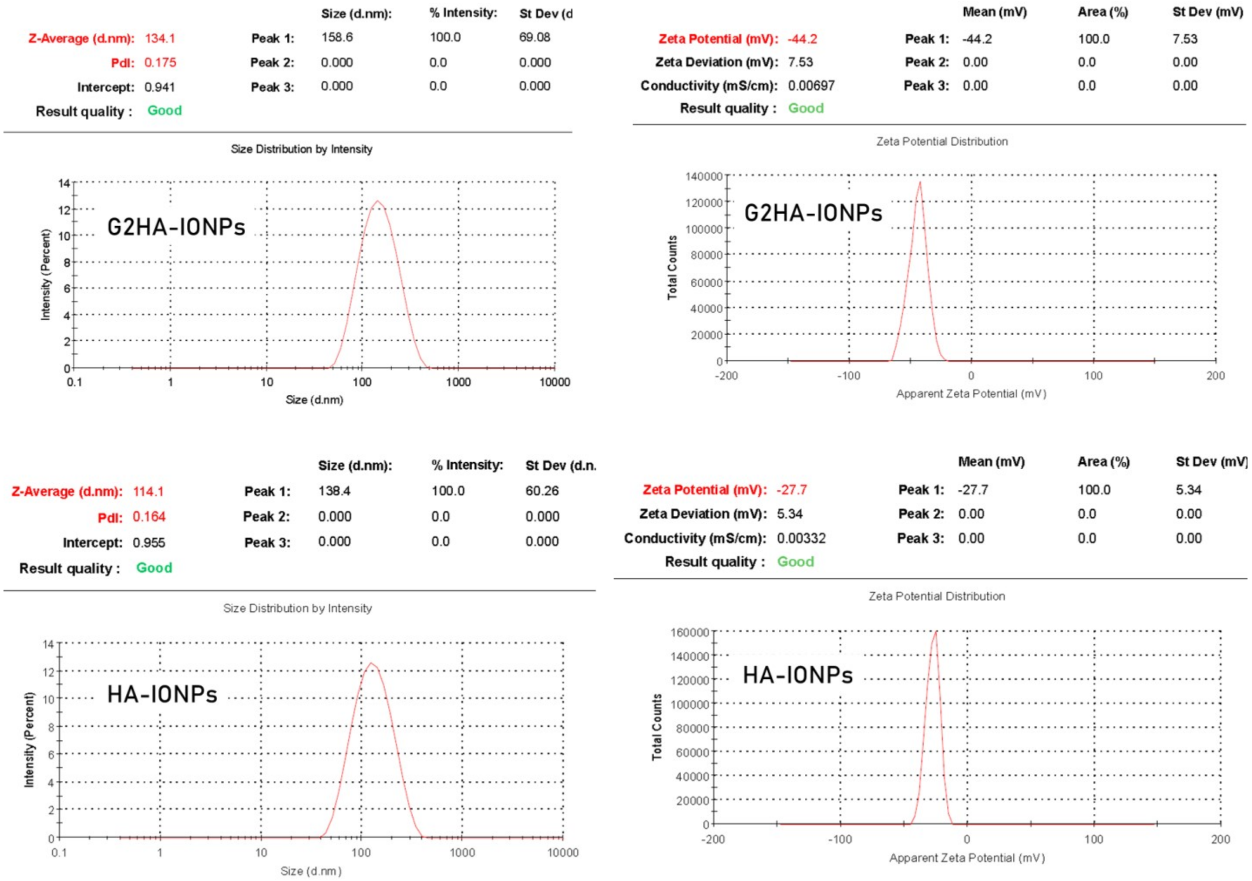

Fig. S2 Hydrodynamic diameter, polydispersity index (PDI), and zeta potential of G2HA-IONPs and HA-IONPs.

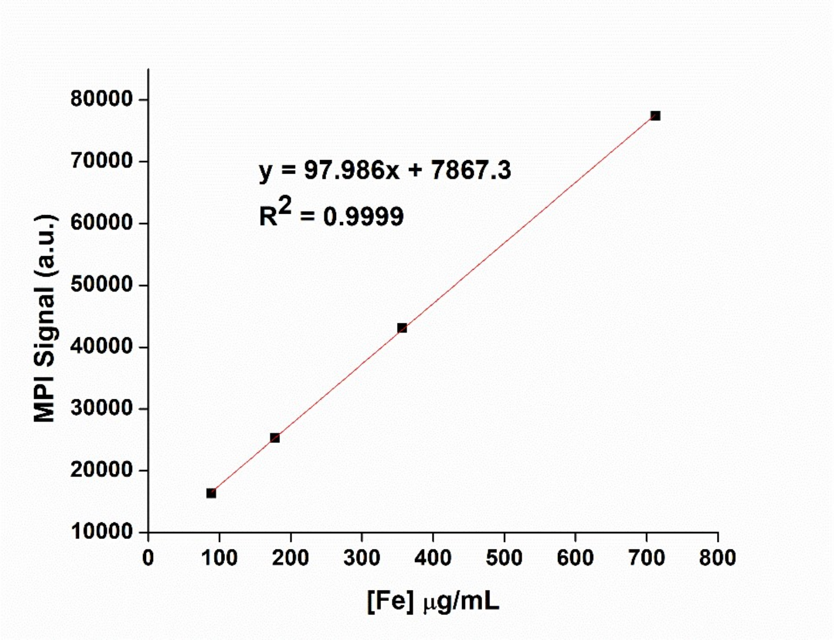

Fig. S3 MPI signal of serial dilutions of HA-IONPs solution showing the linear relationship between iron concentration and the generated MPI signal.

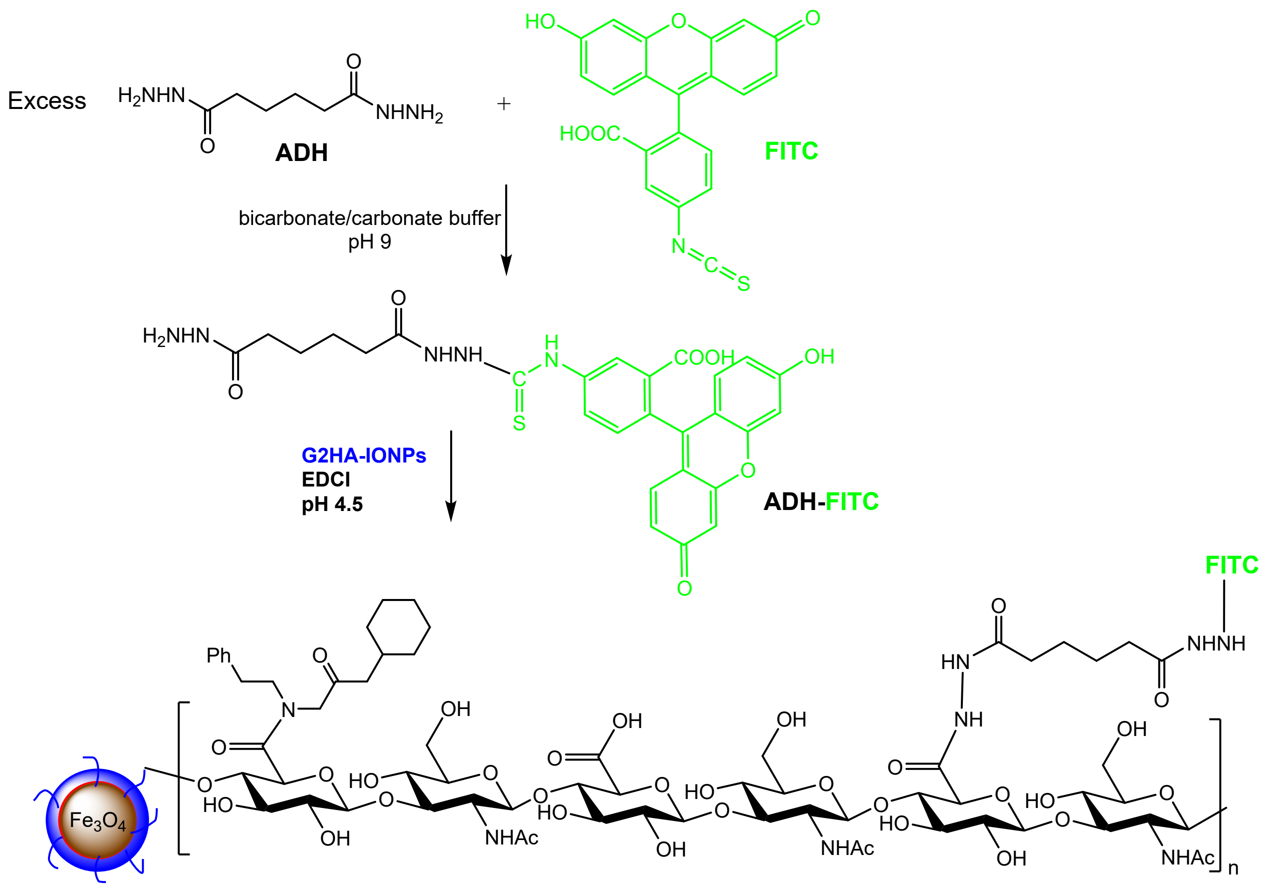

Scheme S1. Synthesis of FITC-labeled G2HA-IONPs.
